# Transforming Dementia Care: Implementation Challenges Moving Evidence-Based Programs to Health Care

**DOI:** 10.1093/geroni/igab046.661

**Published:** 2021-12-17

**Authors:** Laura Gitlin, Kenneth Hepburn, Sara Czaja

## Abstract

Although evidence for dementia care programs continues to grow, families and health providers do not have ready access to programs, nor have they been widely disseminated and routinized in healthcare. Understanding implementation considerations when embedding evidence-based programs in healthcare systems can inform ways to effectively transform dementia care. This symposium will examine similarities and differences in implementation challenges encountered and strategies used when implementing four evidence-based programs being tested in different healthcare environments using distinct study designs. Dr. Gaugler et al., will discuss implementation challenges encountered with a staff-delivered intervention (ADS Plus) to support caregivers in adult day services that is being tested using a mixed methods hybrid trial design in >50 sites nationally. Dr. Hodgson et al., will discuss adaptions and their measurement to COPE, a home-based dyadic support program being embedded in 10 PACEs of a large healthcare system using a noninferiority trial testing staff training strategies. Dr. Forester et al., will examine implementation of the Care Ecosystem for dementia patients in a high-risk, integrated care management program using a pilot embedded pragmatic trial. Dr. Hepburn et al., will explore tactical challenges of implementing Tele-Savvy, an online caregiver psychoeducation program, within the context of a pilot pragmatic clinical trial. Drawing upon implementation science, themes discussed include balancing adaptations and fidelity, measurement of implementation outcomes and organizational readiness, and staff training implications. Also highlighted are research design considerations. Dr. Czaja, an expert in the design and implementation of dementia care interventions from in-person to technology-based will be the discussant.

